# The synergistic protective effects of bioactive catechins in longjing tea: alleviate indomethacin-induced gastric toxicity through modulation of inflammatory and apoptotic pathways

**DOI:** 10.3389/fphar.2026.1828212

**Published:** 2026-05-26

**Authors:** Atilla Topcu, Ayca Toprak-Semiz, Esra Deniz, Cigdem Ozturk, Ihsan Nalkiran, Hatice Sevim Nalkiran, Sibel Mataraci Karakas, Medeni Arpa

**Affiliations:** 1 Department of Pharmacology, School of Medicine, Recep Tayyip Erdogan University, Rize, Türkiye; 2 Department of Pathology, Ministry of Health, Recep Tayyip Erdogan University Training and Research Hospital, Rize, Türkiye; 3 Department of Medical Biology, School of Medicine, Recep Tayyip Erdogan University, Rize, Türkiye; 4 Department of Medical Biochemistry, School of Medicine, Recep Tayyip Erdogan University, Rize, Türkiye

**Keywords:** anti-apoptotic, anti-inflammatory, antioxidant, gastric ulcer, indomethacin, longjing tea

## Abstract

**Objective:**

*Camellia sinensis* L. has for many years been one of the most extensively produced and consumed tea products worldwide. Although interest in Longjing tea (LT) has grown due to its antioxidant and anti-inflammatory properties, studies exploring its potential health effects remain limited. The purpose of this study was to reveal the potential preventive effects of LT on oxidative stress, inflammation, and apoptosis occurring in gastric ulcers induced by indomethacin.

**Methods:**

The composition of LT was determined using HPLC (High-Performance Liquid Chromatography). The control group received only tap water via the oral route for 14 days. The indomethacin-only group was administered 100 mg/kg indomethacin in a single oral dose following 24-h fasting. The indomethacin + famotidine group received 40 mg/kg famotidine via the oral route 1 hour before gastric ulcer induction. The members of the indomethacin + LT group received oral LT for 14 consecutive days. A gastric ulcer was induced after 24-h of fasting, and the experimental groups were sacrificed at the end of the sixth hour. Malondialdehyde, glutathione, cyclooxygenase 1 and 2, vascular endothelial cell growth factor A, nuclear factor kappa B, cleaved Poly ADP-ribose polymerase, pan-Akt, and p-Akt levels were examined at the tissue level.

**Results:**

Oxidative stress and inflammation, increased by indomethacin, activated the apoptotic cascade. LT showed partial anti-oxidative changes but significantly suppressed PARP-1 expression. The limited bioavailability of catechins may have restricted protective efficacy.

**Conclusion:**

The research showed that LT may be a promising agent with partial antioxidant, anti-inflammatory, and anti-apoptotic properties.

## 1 Introduction

Pain is one of the leading health problems associated with a significant decline in quality of life. In addition to various therapeutic approaches developed since ancient times, considerable efforts have been made to identify plant-derived pharmacological agents for pain management (Aboelsoud, 2010; Kovacevic et al., 2024; Shaikh et al., 2025). Non-steroidal anti-inflammatory agents (NSAIDs) began to be widely used in the mid-1800s following advances in chemical isolation techniques, resulting in a breakthrough in the relief of pain and inflammation. However, their increasing use has been associated with the development of gastrointestinal complications, particularly gastric damage.

Gastric ulcers caused by NSAIDs continue to represent an important global health problem. Research has shown that the prevalence of gastric ulcer development in patients using NSAIDs ranges from 10% to 30%, and that increasing age, multidrug use, and secondary diseases further increase this figure among such users (Kovacevic et al., 2024). In addition to acute pain management, long-term use as an anticoagulant or in inflammatory muscle diseases significantly increases the risk of NSAID-induced gastric injury. The principal gastric problems include mucosal erosion in the stomach and submucosal hemorrhage due to tissue damage. The underlying reason NSAIDs lead to gastric injury is the reduction of prostaglandin levels. The non-specific inhibition of cyclooxygenase (COX), which is principally responsible for prostaglandin synthesis, particularly COX-1, by NSAIDs leads to decreased mucus and bicarbonate secretion, suppression of epithelial cell proliferation, and disruption of mucosal blood flow, thereby triggering gastric damage and ultimately ulcer development (Ho S, 2025). Reducing gastric acid secretion is a key therapeutic approach in the treatment of gastric ulcers. Pharmacological antagonism of histamine H_2_ receptors represents one of the principal mechanisms for achieving this effect. Although this represents an effective treatment, the recovery of parietal cell function following withdrawal of H_2_ receptor blockade leads to continued acid secretion (Wang et al., 2025). Moreover, long-term use may result in deficiencies of essential minerals and vitamins, and studies have also suggested a possible dose-dependent association with an increased risk of cancer (Sawaid and Samson, 2024). Therefore, the development of protective or preventive strategies in addition to existing treatment options for gastric disorders that significantly impair quality of life, has become an important area of research.

The investigation of plant-based solutions in the treatment of gastric pathologies has become increasingly popular in recent years. In parallel with the development of new-generation synthetic molecules and drugs, interest in organic and plant-derived therapies with more tolerable side effects has also increased over the last century. There has also been a shift toward traditional treatments, such as Chinese medicine, partly due to concerns over adverse effects associated with conventional drug use. In addition to being a component of social culture, tea is frequently consumed as a daily beverage and is widely recognized for its beneficial effects, making it a subject of growing research interest. The beneficial health effects of herbal teas are well-documented and similar effects have been observed for tea itself as a widely consumed and culturally significant beverage. Studies have also suggested its potential role as a preventive agent. Previous studies have shown that green tea, in particular, exhibits cancer-preventive, antioxidant, and anti-inflammatory properties (Deng et al., 2025). Longjing tea (Dragon Well tea) examined in the present study, is an important variety of green tea. Previous studies have reported that it possesses significant antioxidant capacity with potential medical benefits (Yashin et al., 2011; Lv et al., 2022). Research has shown that (-)-epigallocatechin3-gallate (EGCG), one of the major polyphenolic compounds in this tea, may exert beneficial effects on NSAID-induced gastric ulcers. It has been reported EGCG prevents indomethacin-induced injury by increasing prostaglandin levels and accelerating the healing process (Adhikary et al., 2011). Through the synergistic effect of its bioactive compounds, Longjing tea may exhibit a protective effect against tissue damage. However, the oxidative stress, inflammatory, and apoptotic pathways underlying these effects remain incompletely understood.

This study was performed to investigate the potential protective and preventive effects of Longjing tea by enhancing antioxidant, anti-inflammatory, and antiapoptotic responses in gastric tissue.

## 2 Materials and methods

### 2.1 Chemicals

Longjing tea was obtained from teaChef Trade Co. Ltd., Rize, Türkiye. Compliance with the relevant standards was verified. Indomethacin (Endol 25 mg capsule) was obtained from Deva Holding A.Ş., Istanbul, Türkiye, and famotidine (Famodin 40 mg film tablet) from Sandoz İlaç San. ve Tic. A.Ş., Istanbul, Türkiye. Anesthesia was induced using ketamine hydrochloride (Ketalar, 500 mg/10 mL, Pfizer İlaçları Ltd. Şti., Istanbul, Türkiye) and xylazine hydrochloride (Rompun, 2%, 25 mL, Bayer Türk Kimya San. Ltd. Şti., Istanbul, Türkiye). All chemicals used in the laboratory experiments were obtained from Sigma Chemical Co. and Merck (Germany).

### 2.2 High-performance liquid chromatography (HPLC) and total polyphenol analysis

Total catechin and polyphenol ingredients analyses were carried out according to TS EN ISO/IEC standards by the Rize Food Control Laboratories Directorate using HPLC (Shimadzu RID-20A, Japan) and a detector (HACH LANGE GmbH, Germany), respectively.

HPLC analysis was performed as follows: briefly, the prepared extraction solution was incubated in a water bath at 70 °C. Once the sample had been brought to room temperature, it was centrifuged at 3500 r/min for 10 min. After adding 5 mL of 70 °C methanol extraction mixture, the sample was subjected to stepwise incubation. It was again cooled to room temperature, and centrifuged at 3500 r/min for 10 min. One milliliter of the prepared extract was transferred to a tube and placed in a 5 mL measuring flask, filled with the stabilized solution, and mixed using a magnetic stirrer. The diluted sample was filtered through a 0.45 µm filter into an HPLC vial and analyzed by HPLC. Total polyphenol measurement was performed as follows: the extraction solution was equilibrated in a 70 °C water bath for 30 min. Then, 5 mL of extraction solution was added to the centrifuge tube containing the sample and vortexed thoroughly for complete extraction. The mixture was centrifuged at 3500 rpm for 10 min. Subsequently, 5 mL of the 70 °C methanol extraction mixture was added to the pellet and mixed again for 15 s. The supernatant was carefully removed. Cold extraction solution was added, and 1 mL of the prepared extract was transferred to a 100 mL volumetric flask, which was then filled with distilled water. One milliliter of the diluted extract was then transferred to a 10 mL centrifuge tube, and 5 mL of Folin reagent was added and mixed. The solution was incubated in the dark at room temperature for 60 min, during which it developed a bluish color. Finally, absorbance was measured at 765 nm using a spectrophotometer against distilled water as the blank (Supplementary Material).

### 2.3 Experimental animals

All experimental animal procedures were performed in accordance with the ARRIVE 2.0 guidelines describing animal research published in PLOS Biology on July 2020 (du Sert et al., 2020). Twenty-eight male Sprague Dawley rats, 8 weeks of age and weighing 280-300 grams, were used in this study. All procedures involving experimental animals were carried out in accordance with the Guide for the Care and Use of Laboratory Animals and relevant national and international regulations. Before and during the procedures, all animals were housed in standard transparent polycarbonate cages with sawdust bedding, at a temperature of 20–24 °C and 55%-65% humidity, under a 12-h light/dark cycle. The rats had *ad libitum* access to standard chow and tap water prior to the study, while only tap water was provided during the experimental period. The study was conducted following approval by the Recep Tayyip Erdoğan University Animal Experiments Ethics Committee (approval no. 2025/07-07.02.2025).

### 2.4 Experimental protocol and application of the indomethacin-induced gastric ulcer model

Rats were randomly divided into four groups of seven animals each, with similar average weights.

All rats were fasted for 24 h before gastric ulcer induction, with only *ad libitum* access to water being permitted. Group 1, the control (C) group, received only tap water orally for 14 days. Group 2 was the indomethacin (INDO) only group. Members of this group were given a single oral dose of 100 mg/kg indomethacin following a 24-h fast and were sacrificed at the end of the sixth hour (Aydin et al., 2025). Group 3, the INDO + Famotidine (FAM) group (INDO + FAM), received 40 mg/kg FAM orally 1 hour before gastric ulcer induction, and these rats were also sacrificed at the end of the sixth hour. Group 4, the INDO + Longjing tea (LT) (INDO + LT) group received oral Longjing tea for 14 consecutive days. Rats were provided with LT, freshly prepared as a hot-water infusion and allowed to cool to room temperature, as their sole drinking fluid *ad libitum* for 14 days (≈ 2-4 g/500 mL). In healthy adult rats, daily fluid intake typically ranges between 20–35 mL. In a previous study by Daily JW. et al., it was reported as 30.8 ± 2.2 mL (Daily et al., 2019). Based on this intake, the estimated daily LT consumption was approximately 180 mg per rat, administered as a hot-water infusion cooled to room temperature. Sato H et al. reported that administration of catechin-containing drinking water for 14 days suppressed gastrin secretion from G cells and exerted a direct modulatory effect on enterochromaffin-like (ECL) cells in rats (Sato et al., 2002). Accordingly, this study was designed to evaluate whether 14-day administration of the natural product LT exerts measurable anti-inflammatory, antioxidant, and anti-apoptotic effects, consistent with the reported biological activities of catechin-rich compounds. On day 14, following a 24-h fast, a single dose of 100 mg/kg indomethacin was administered orally, and all animals were sacrificed 6 hours later using high-dose anesthesia. Stomach tissues were collected, with one portion stored at −86 °C for biochemical and molecular analyses and the other fixed 10% neutral formalin.

### 2.5 Biochemical procedures

#### 2.5.1 Tissue sampling and homogenization

Blood was first removed from the tissue specimens by washing with phosphate-buffered saline (PBS, pH: 7.4). Phosphate buffer was then added such that the volume would be double the weight. The tissues were then homogenized using a 30 Hertz/5 min homogenizer (TissueLyser II, QIAGEN, Germany). Finally, they were centrifuged at 3000 × g for 15 min, and the resulting supernatants were used for biochemical analyses (Topcu et al., 2023).

#### 2.5.2 Measurment of malondialdehyde (MDA), glutathione (GSH) and vascular endothelial growth factor A (VEGF-A) levels in gastric tissue homogenates

MDA, GSH, and VEGF-A levels in gastric tissue homogenates were measured using rat-specific ELISA kits (catalog numbers E-EL-0060, E-EL-0026, and E-EL-R2603, respectively, Elabscience, Houston, Texas, USA) (Ansari et al., 2025; Aydin et al., 2025). The sensitivity values were 18.75 ng/mL, 0.94 μg/mL, and 18.75 pg/mL, respectively. Detection range values were 31.25–2000 ng/mL, 1.56-100 μg/mL, and 31.25-2000 pg/mL, respectively. Tissue homogenates were prepared in accordance with the manufacturer’s instructions.

#### 2.5.3 Cyclooxygenase-1 (COX-1) and Cyclooxygenase-2 (COX-2) analysis

COX-1 and COX-2 levels in rat gastric tissue homogenates were measured using rat-specific ELISA kits (catalog numbers CSB-E13416r and CSB-E13399r, respectively, Cusabio, Wuhan, China) (Alfadil, 2024; Bolat et al., 2025). Detection range values were 7.8 pg/mL–500 pg/mL, and 1.56 ng/mL–100 ng/mL, respectively. Tissue homogenates were prepared in accordance with the manufacturer’s instructions.

### 2.6 Western blotting

The tissue samples were homogenized with RIPA buffer containing protease inhibitors and centrifuged for 15 min at 14,000 rpm at 4 °C. The quantities of protein in the supernatant parts were determined using a Pierce Dilution-Free Rapid Gold BCA protein assay kit (Thermo Fisher Scientific, Waltham, MA, USA; Cat. No. A55861). Next, 25 µg protein was taken from each sample and denatured with Laemmli Sample Buffer (2x) (Ecotech Biotechnology, Erzurum, Türkiye; Cat. No. LSB-2x). The proteins were then separated on 10% SDS-PAGE gel and transferred to PVDF membranes (GVS North America, USA; Cat. No. 1212639). The membranes were blocked with TBST and incubated overnight at 4 °C with PARP1 (cleaved Asp214, Asp215) (1:1000) (Thermo Fisher Scientific, Waltham, MA, USA; Cat. No. 44-698G) and GAPDH (1:100000) (Abclonal Technology, Woburn, USA; Cat. No. AC033) primary antibodies (He et al., 2019). After washing, the membranes were incubated with HRP conjugated Anti-Mouse IgG (HRP) (Cell Signaling Technology, MA, USA; Cat. No. 7076S) and Goat Anti-Rabbit IgG H&L (HRP) (Abcam, Cambridge, United Kingdom; Cat. No. ab205718) secondary antibodies. Signals were developed with Clarity Western ECL Substrate (Bio-Rad Laboratories, Hercules, CA, USA; Cat. No. 1705060) and visualized using the ChemiDoc Imaging System (Bio-Rad Laboratories, Hercules, CA, USA). Band intensities were analyzed using ImageJ software (National Institutes of Health, Bethesda, MD, USA) and normalized to GAPDH. The experiments were conducted independently in triplicate. All analyses were based on the means of three independent experiments, with significance levels of *p < 0.05 and ***p < 0.001.

### 2.7 Histopathological analysis

#### 2.7.1 Macroscopic and ulcer index score analysis

Gastric tissue samples were removed, washed with saline, and excess saline was carefully removed. The inner surface of the stomach was then opened and photographed using a digital camera. Mucosal lesions, including inflammatory and hemorrhagic areas, were evaluated macroscopically. The percentage of hemorrhagic areas was calculated using counting paper. The following formula was used to calculate the ulcer index:

*Ulcer index: 10 X Total ulcerated area/Total mucosal area* (AlKreathy et al., 2020).

#### 2.7.2 Microscopic histopathological analysis

Gastric tissue samples were cut into approximately 1.5 cm^3^ pieces and fixed in 10% neutral buffered formalin solution for 24 h. The samples were then dehydrated through a graded ethanol series and cleared in xylene before being embedded in paraffin. Sections 4 µm in thickness were cut from paraffin blocks using a rotary microtome (ASP300S, Leica, Germany). Tissue sections were stained with Harris hematoxylin and eosin (Beslab, Türkiye). Gastric tissues from each rat were examined by an experienced histopathologist blinded to the experimental groups. Histopathological damage scores were determined using the scoring system proposed by AlKreathy et al. (2020), Alamri (2024). Histopathological changes were evaluated based on the severity of gastric damage as follows: epithelial cell loss (score: 0–3), hemorrhage (score: 0–4), inflammatory cell infiltration (score: 0–2) and mucosal erosions (score: 0–4). The total histopathological damage score was calculated by summing the scores of each parameter and compared among the groups.

#### 2.7.3 Immunohistochemical (IHC) analysis

Nuclear factor kappa B (NF-κB/p65, BS-20159R, Bioss Antibodies Inc. Massachusetts, USA), vascular endothelial growth factor A (VEGF-A) (anti-VEGF-A; AF5131, Affinity Biosciences, Victoria, Australia), pan-AKT (anti-pan-AKT; AF6261, Affinity Biosciences, Victoria, Australia), and phospho-Akt (p-Akt) (anti-p-Akt, ser 473 AF0016, Affinity Biosciences, Victoria, Australia), were used (Ding et al., 2024; Liu et al., 2026). All procedures were conducted automatically in accordance with the recommendations of the manufacturer of the primary antibodies, on a Ventana BenchMark Special Stains System (Roche Diagnostics Türkiye Inc., Istanbul, Türkiye). Human placenta tissue was used as an external control for VEGF-A and p-AKT, while human tonsil tissue served as a positive control for pan-AKT and NF-κB/p65.

#### 2.7.4 Semi-quantitative analysis

The immunoreactive score (IRS) method developed by Remmele W. et al. (1987) was used to evaluate immunohistochemical staining (Remmele W, n.d.). Areas incubated with primary antibodies from each sample were evaluated by a pathologist blinded to the study groups. In this system, each stained section was evaluated based on staining intensity and staining percentage. IRS; 0 represented a zero IHC positivity score (0%-1%); 1 represented weak positivity (<10%); 2 represented moderate positivity (11%–50%), 3 represented severe positivity (51%–80%), and 4 represented over-severe positivity (>80%).

### 2.8 Statistical analysis

The biological parameters (MDA, GSH, VEGF-A, COX-1, and COX-2) and histopathological scores were analyzed for group comparisons. The Kruskal-Wallis test was employed to assess group-level differences, and pairwise comparisons for significant parameters were conducted using the Bonferroni-corrected Mann-Whitney U test. The data are presented as medians (interquartile range: IQR). All statistical analyses were performed using IBM SPSS Statistics version 29.0 software (IBM Corp., Armonk, NY, USA), *p* < 0.05 was considered statistically significant.

## 3 Results

### 3.1 Identification of putatively active compounds in longjing tea using HPLC

The catechin levels in Longjing tea were determined using HPLC. The analysis revealed a high level of catechin content, which represents a key bioactive component of tea known for its antioxidant, anti-inflammatory, and antiapoptotic properties (Table 1).

**TABLE 1:** Characterizations of polyphenol compounds present in Longjing tea using HPLC analysis.

ID#	Name	RET.TIME	Area	CONC.	Units
1	Gallic acid	5.131	285415	8.237	ppm
2	Epigallocatechin (EGC)	9.008	102923	37.156	ppm
3	Catechin (C)	12.331	1087192	564.740	ppm
4	Caffeine	15.026	3922154	118.957	ppm
5	Epicatechin (EC)	16.074	140106	15.834	ppm
6	Epigallocatechin gallate (ECGG)	19.524	51008114	344.298	Ppm
7	Epicatechin gallate	24.343	2009933	91.296	Ppm

### 3.2 Biochemical analysis results

#### 3.2.1 Analysis of MDA, GSH and VEGF-A levels

MDA levels, a marker of lipid peroxidation, reflected the oxidative stress response in the indomethacin-induced gastric ulcer model. A slight increase was observed in the INDO group compared to the control group, although this was not statistically significant (*p* = 0.234). Median tissue MDA levels were lowest in the group given famotidine together with indomethacin (INDO + FAM), and differed significantly from the INDO and INDO + LT groups (*p* = 0.001 and *p* < 0.001, respectively, Table 2). These findings show that famotidine is capable of suppressing lipid peroxidation more effectively than Longjing tea. Although a slight decrease was observed in the INDO + LT group compared to the control and INDO groups, this was not statistically significant (*p* = 1.000 and *p* = 0.308, respectively). GSH levels, an important marker of cellular antioxidant capacity, were used to evaluate the response to the treatment applied in the experimental ulcer model. A mild increase in GSH levels was observed in the indomethacin group compared to the control group, although this was not statistically significant (*p* = 0.122). A marked increase was observed in the INDO + FAM group compared to the INDO group, although this was also insignificant (p = 0.768). The significant difference observed compared to the control group (*p* = 0.041) was no longer significant after Bonferroni correction (*p* = 0.122). A slight increase was observed in the INDO + LT group compared to the indomethacin only group, but this was again not significant (*p* = 0.656); the significant elevation compared to the control group (*p* = 0.027) also disappeared following Bonferroni correction (*p* = 0.081). Comparable GSH levels were detected in the INDO + FAM and INDO + LT groups (*p* = 0.635). This showed that famotidine and Longjing tea may contribute to enhancing cellular antioxidant capacity.

**TABLE 2:** Biochemical parameter levels among the experimental groups.

Biochemical parameters	Control	INDO	INDO + FAM	INDO + LT
VEGF-A (pg/g tissue)	2558 (916)	2214 (1836)	5046.5 (5765)^b^	1925.5 (799)
COX-1 (pg/g tissue)	252 (115)	169 (74)	708 (532)^c^	496 (1113)^d^
COX-2 (ng/g tissue)	209.2 (226.8)	58 (155.9)	333.3 (320)	133.25 (39.3)
MDA (ng/g tissue)	1121 (767)	1265 (1009)	158.5 (355)^a^	1105.5 (280)
GSH (ug/g tissue)	2.71 (2.54)	3.9 (1.25)	6.01 (2.13)	6.39 (6.26)

a *p* = 0.001, *p* < 0.001; respectively, compared to the INDO, and INDO + LT, group

b *p* = 0.011, compared to the INDO + LT group

c *p* = 0.006, compared to the INDO group

d *p* = 0.009, compared to the INDO group

Mann-Whitney U test with Kruskal-Wallis/Bonferroni correction.

A slight decrease was observed in median tissue VEGF-A levels following indomethacin administration compared to the control group (*p* = 0.721), although this was not statistically significant. The highest VEGF-A levels were determined in the group receiving famotidine in combination with indomethacin (INDO + FAM). Although this increase was not significant compared to the control (*p* = 0.815) or INDO (*p* = 0.078) groups, it was statistically significant compared to the INDO + LT group (*p* = 0.011, Table 2). This shows that famotidine stimulates a more powerful angiogenic response than Longjing tea. Median tissue VEGF-A levels were lowest in the INDO + LT group, but showed no significant difference compared to the control or INDO groups (*p* = 0.327 and *p* = 1.000, respectively). This suggests that Longjing tea may exert a pronounced lowering effect on VEGF-A levels.

#### 3.2.2 COX-1 and COX-2 analysis results

Examination of COX-1 levels revealed that indomethacin administration resulted in a decrease in median tissue values compared to the control group, although this was not statistically significant (*p* = 0.896). Median COX-1 levels in the group receiving indomethacin with famotidine increased significantly compared to the INDO group (*p* = 0.006, Table 2). However, that increase was not significant compared to the control and INDO + LT groups (*p* = 0.106 and *p* = 1.000, respectively). Significantly higher COX-1 levels were determined in the group receiving indomethacin together with Longjing tea (INDO + LT) compared to the indomethacin only group (*p* = 0.009, Table 2). However, that increase was not significant compared to the control group (*p* = 0.232). These findings suggest that both famotidine and Longjing tea may exert a protective or enhancing effect on COX-1 levels in gastric tissue. Indomethacin administration caused a decrease in COX-2 levels compared to the control group, although this did not reach statistical significance (*p* = 0.526). The highest median tissue COX-2 levels were observed in the INDO + FAM group; however, no significant differences were found compared to the control and INDO + LT groups (*p* = 0.128 and *p* = 0.093, respectively). This suggested that famotidine may partially increase COX-2 levels. A slight increase was also observed in the INDO + LT group compared to the INDO group, although this difference was not significant (*p* = 1.000).

### 3.3 Western blot analysis results

**FIGURE 1:**
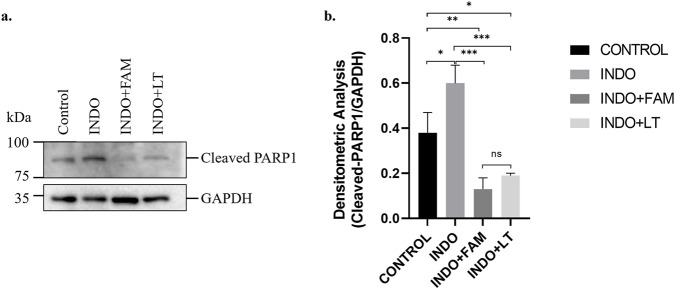
**(a)** Western blot analysis of cleaved PARP1 in tissue samples. GAPDH was used as a loading control. **(b)** Densitometric analysis of cleaved PARP1/GAPDH ratios. Data are presented as the mean ± standard deviation (SD) from three independent experiments (*p < 0.05, **p < 0.01, ***p < 0.001). ns indicates a non-significant difference. The experimental groups were as follows: Control, Indomethacin (INDO), Indomethacin + Famotidine (INDO + FAM), and Indomethacin + Longjing tea (INDO + LT).

Western blot analysis revealed a significant difference among the treatment groups in terms of cleaved PARP-1. At densitometric evaluation, the cleaved PARP-1/GAPDH ratio in the control group was approximately 0.35, while the application of indomethacin raised that value significantly to 0.65 (*p < 0.05, Figure 1). This increase shows that indomethacin significantly stimulates the activation of cleaved PARP-1. The addition of famotidine to the indomethacin application (INDO + FAM) resulted in a significant decrease in cleaved PARP-1 compared to the INDO group (***p < 0.001, Figure 1). Indomethacin in combination with Longjing tea (INDO + LT) also reduced cleaved PARP-1 expression in a more marked manner (***p < 0.001, Figure 1). No statistically significant difference was determined between the INDO + FAM and INDO + LT groups. This showed that both applications resulted in similar suppression of cleaved PARP-1 activation induced by indomethacin. Overall, the findings show that indomethacin increased cleaved PARP-1 activation, whereas famotidine and Longjing tea were associated with reduced activation, suggesting a potential protective effect.

### 3.4 Histopathological analysis results

**FIGURE 2:**
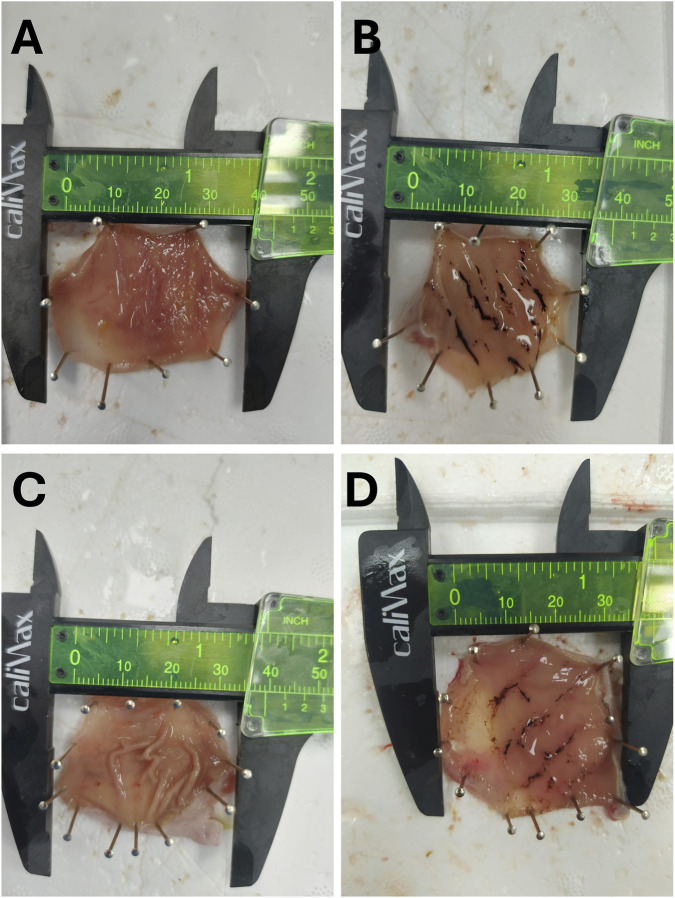
Macroscopic appearance of gastric mucosa. **(A)** The control **(C)** group. **(B)** The indomethacin (INDO) group, hemorrhagic areas in the mucosa. **(C)** A sample from the INDO + Famotidine (INDO + FAM) group, no hemorrhage was macroscopically visible in the mucosa. **(D)** An example from the INDO + Longjing tea (INDO + LT) group, hemorrhage in focal areas of the mucosa.

**FIGURE 3:**
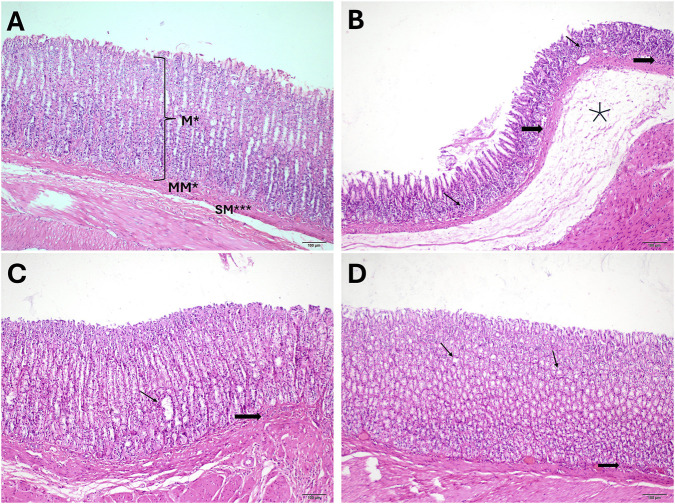
Microscopic appearance of gastric mucosa. **(A)** (X10): A sample from the control (C) group exhibiting a normal structure in the gastric mucosa (M*: Mucosa, MM*: Muscularis mucosa, SM***: Submucosa). **(B)** (X10): A section from the indomethacin (INDO) group exhibiting disruption of mucosal integrity and increased inflammatory cell infiltration (Thin arrow: Damage to glandular structures in the mucosa; Thick arrow: Inflammatory cell infiltration, Star: Submucosal edema and hemorrhage). **(C)** (X10): A section from the INDO + Famotidine (INDO + FAM) group with minimal structural change in the mucosa and mild inflammatory infiltration (Thin arrow: Damage to glandular structures in the mucosa, Thick arrow: Inflammatory cell infiltration). **(D)** (X10): Section from the INDO + Longjing tea (INDO + LT) group exhibiting a regular mucosal structure and minimal inflammatory infiltration (HE x100) (Thin arrow: Damage to gland structures in the mucosa, thick arrow: Inflammatory cell infiltration).

Macroscopic and light microscopic examination of gastric tissues from the control group revealed a normal appearance, with a healthy mucosal structure, as well as the absence of hemorrhagic lesions in the muscularis mucosa and submucosa (Figures 2, 3). In contrast, in the INDO group, an extensive gastric area with impaired mucosal integrity, increased inflammatory cell infiltration, and pronounced hemorrhage in the mucosal area, together with submucosal edema were observed (Figure 3; Table 3). However, minimal structural changes and mild inflammatory cell infiltration were present in the INDO + FAM group. In the INDO + LT group, a decreased musical gastric surface, a more regular mucosal area, and less inflammatory cell passage were observed compared to the INDO group (Figure 3; Table 3). These findings showed that Longjng tea has the potential to exhibit protective effects at the cellular level.

**TABLE 3:** Macroscopic and microscopic appearance of the gastric mucosa.

Histopathological findings	Control	INDO	INDO + FAM	INDO + LT
	Median (min-max)	Median (min-max)	Median (min-max)	Median (min-max)
Mucosal erosion	1 (0-1)	3 (1-4)^a^	1 (1-2)	1 (0-1)^b^
Inflammatory cell infiltration	0 (0-1)	2 (2-2)^c^	1 (0-1)^d^	1 (0-2)^e^
Hemorrhage and necrosis	0 (0-1)	3 (2-4)^f^	1 (0-1)	1 (0-2)^g^
Epithelial cell loss	0 (0-1)	3 (2-3)^h^	1 (0-2)	1 (0-1)^i^
Ulcer%	0 (0-0)	30 (15-30)^J^	0 (0-0)^k^	22.5 (5-35)^l^
Ulcer index	0 (0-0)	3 (1.5-3)^m^	0 (0-0)^n^	2.25 (0.5-3.5)^o^

a *p* = 0.001. Compared to the C group

b *p* = 0.015, compared to the INDO group

c *p* = 0.000, compared to the C group

d *p* = 0.010, compared to the INDO group

e *p* = 0.035, compared to the INDO group

f *p* = 0.001, compared to the C group

g *p* = 0.022, compared to the INDO group

h *p* = 0.000, compared to the C group

i *p* = 0.034, compared to the INDO group

j *p* = 0.000, compared to the C group

k *p* = 0.001, compared to the INDO group

l *p* = 0.004, compared to the C group

m *p* = 0.000, compared to the C group

n *p* = 0.001, compared to the INDO group

o *p* = 0.003, compared to the C group

Mann-Whitney U test with Kruskal-Wallis/Bonferroni correction.

#### 3.4.1 Immunohistochemical (IHC) analysis results

**FIGURE 4:**
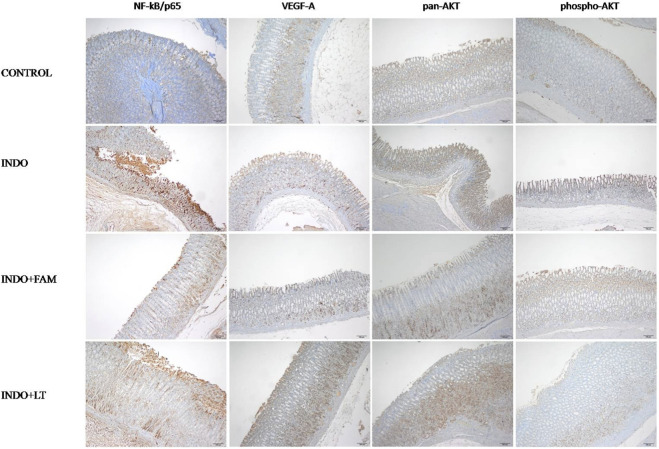
Representative light microscopy images of immunohistochemistry-stained gastric mucosal tissue sections. Control group (x100): Diffuse, low-intensity cytoplasmic immunoreactivity with Nuclear factor kappa B (NF-κB/p65); Low-to-moderate diffuse immunoreactivity with Vascular endothelial growth factor A (anti-VEGF-A); Diffuse and low-intensity cytoplasmic immunoreactivity with pan-AKT (anti-pan-AKT); Mild to moderate immunoreactivity with phospho-Akt (p-Akt) (anti-p-Akt) primary antibody in healthy mucosa. INDO group (x100): Severe cytoplasmic and nuclear immunoreactivity with NF-κB/p65; Moderate-to-severe immunoreactivity with VEGF-A; Moderate to occasionally severe immunoreactivity with pan-AKT; Moderate to occasionally severe immunoreactivity with p-Akt in the INDO-damaged mucosa. INDO + FAM group (x100): Moderate-to-severe diffuse immunoreactivity with NF-κB/p65; Occasionally severe immunoreactivity with VEGF-A; Locally severe immunoreactivity with pan-AKT; Widespread low-to-moderate immunoreactivity with p-Akt in a healing mucosa. INDO + LT group (x100): Moderate-to-severe diffuse immunoreactivity with NF-κB/p65; Diffuse-to-moderately severe immunoreactivity with VEGF-A; Severe immunoreactivity in some places with pan-AKT; Widespread moderate to occasionally severe immunoreactivity with p-Akt in the gastric mucosa samples.

Gastric tissue sections from all groups were stained with primary antibodies against NF-κB/p65, VEGF-A, pan-AKT, and p-Akt. IHC analysis revealed varying degrees of NF-κB/p65, VEGF-A, pan-AKT, and p-Akt positivity among the groups. Evaluation of expression levels showed a significant increase in NF-κB/p65 positivity percentage, score, and severity in the INDO group compared to the control group (Figure 4; Table 4; *p* = 0.002, *p* = 0.016, and *p* = 0.005, respectively). A marked increase in the expression of other parameters was also observed, although this did not reach statistical significance. In the INDO + FAM group, NF-κB/p65 severity and p-Akt positivity percentage were significantly decreased compared to the INDO group (Figure 4; Table 4; *p* = 0.015 and *p* = 0.006, respectively). Although changes in expression levels were observed in the INDO + LT group compared to the INDO group, these were not statistically significant. However, NF-κB/p65 positivity percentage, as well as pan-AKT positivity percentage and severity, were significantly higher in the INDO + LT group compared to the control group (Figure 4; Table 4; *p* = 0.002, *p* = 0.008, and *p* = 0.029, respectively). Additionally, although NF-κB/p65 and p-Akt expression levels decreased compared to the INDO group, these decreases were not statistically significant.

**TABLE 4:** Immunohistochemical analysis results.

Immunohistochemical finding		Control	INDO	INDO + FAM	INDO + LT
		Median (min-max)	Median (min-max)	Median (min-max)	Median (min-max)
NF-κB/p65	%	20 (10-30)	60 (30-90)^a^	25 (10-70)	40 (30-70)^b^
	Score	2 (1-2)	3 (2-4)^c^	2 (1-3)	2 (2-3)
	Severity	1 (1-2)	3 (2-3)^d^	1 (1-3)^e^	2 (1-3)
VEGF-A	%	80 (30-90)	80 (70-90)	65 (10-80)	75 (60-80)
	Score	3 (2-4)	3 (3-4)	3 (1-3)	3 (3-3)
	Severity	2 (1-3)	3 (2-3)	1,5 (1-3)	3 (1-3)
Pan-AKT	%	40 (10-70)	60 (30-90)	70 (60-90)	80 (70-80)^f^
	Score	2 (1-3)	3 (2-4)	3 (3-4)	3 (3-3)
	Severity	1 (1-2)	1 (1-3)	2 (1-3)	2.5 (1-3)^g^
p-Akt	%	80 (40-90)	90 (70-90)	25 (20-70)^h^ ^,^ ^i^	75 (70-90)
	Score	3 (2-4)	4 (3-4)	2 (2-3)	3 (3-4)
	Severity	1 (1-2)	2 (1-3)	1.5 (1-3)	1 (1-3)

a *p* = 0.002, compared to the C group

b *p* = 0.040, compared to the C group

c *p* = 0.016, compared to the C group

d *p* = 0.005, compared to the C group

e *p* = 0.015, compared to the INDO group

f *p* = 0.008, compared to the C group

g *p* = 0.029, compared to the C group

h *p* = 0.047, compared to the C group

i *p* = 0.006, compared to the INDO group.

## 4 Discussion

In this study, the design of the experimental groups was established based on previously published studies. Similarly, in line with the approach employed in a previous study conducted by Danisman B. et al., it was demonstrated that the relationships among the variables addressed in the present study could be clearly and reliably elucidated (Danisman et al., 2023).

The dose of the natural product used in this study was selected based on established toxicological and safety data for green tea catechins, particularly EGCG, the most abundant and bioactive catechin found in green tea and a major constituent of LT. Given that the test material is a natural green tea derivative, its safety assessment aligns with the subchronic toxicity data reported in the literature. Previous studies have demonstrated that EGCG is well tolerated in rats at doses up to 500 mg/kg/day, indicating a favorable safety margin. The estimated daily intake in the present study remained substantially below these thresholds, supporting the selection of a non-toxic and physiologically relevant dose (Isbrucker et al., 2006). In addition, hepatotoxicity has been associated primarily with high-dose exposure to green tea catechins, particularly at daily EGCG intakes of 800 mg or higher (Dekant et al., 2017). In contrast, traditionally prepared green tea infusions have consistently shown a favorable safety profile. Consistent with previous assessments by Dekant W. et al., the preparation used in this study, a traditionally prepared green tea aqueous infusion, is expected to demonstrate a similar safety profile (Dekant et al., 2017). Regarding human relevance, the safety of green tea consumption may vary by route of intake. However, conventional aqueous infusions, such as those used in this study, are generally considered safe and are widely consumed (Hu et al., 2018). As the present study used an aqueous preparation rather than a concentrated extract, the estimated intake is considered to fall within the range of conventional consumption. Accordingly, the dosing regimen applied in this study is both biologically relevant and consistent with existing human safety data.

The present study employed biochemical, molecular, and histopathological methods to evaluate the protective effects of Longjing tea in gastric injury induced by high-dose indomethacin, an important NSAID. The findings showed that high-dose indomethacin led to mucosal damage in gastric tissue, thus increasing oxidative stress, inflammation, and apoptosis. In contrast, Longjing tea exhibited beneficial, albeit only partial, effects in preventing gastric injury. These findings are similar to those of recent studies but also exhibit some differences.

Although catechins, which are abundant in LT, have well-documented antioxidant and anti-inflammatory properties, the protective effects observed in high-dose INDO-induced gastric injury models remain limited. This situation can be explained primarily by pharmacokinetic and bioavailability-related factors, including low oral bioavailability of catechins, their chemical instability in the gastrointestinal environment, rapid biotransformation, and limited distribution to target tissues (Tsouh Fokou et al., 2025). Indeed, these constraints reduce the ability of catechins to reach sufficient concentrations in the gastric mucosa, thereby limiting their potent antioxidant and anti-inflammatory effects observed *in vitro*. Furthermore, high-dose INDO exposure may overwhelm endogenous and exogenous protective mechanisms, thereby attenuating the measurable biochemical response. In this context, the biochemical findings of the present study suggest that the observed effects of LT represent a partial therapeutic effect rather than a complete protective outcome.

Previous studies have revealed that indomethacin-induced toxicity in gastric tissue results in an increase in the production of proinflammatory cytokines that trigger reactive oxygen species (ROS) and inflammation mediated by more than one mechanism (Shokati Sayyad et al., 2024; Ansari et al., 2025). The present study evaluated two important parameters for determining oxidative stress levels. One was MDA, the end product of lipid peroxidation and a reactive aldehyde that increases with tissue damage. The other was GSH, which exhibits physiological antioxidant effects against oxidative stress. Previous studies involving indomethacin have similarly shown that MDA concentrations increase with the severity of injury, while GSH levels decrease (Aboelmagd et al., 2025; Ansari et al., 2025). In the present study, MDA levels in gastric tissue increased slightly, while GSH levels decreased slightly in response to this statistically insignificant rise. The increase in MDA concentrations is consistent with the literature, whereas the observed trend in GSH levels suggests that the physiological antioxidant defense system may be activated during the acute phase. In contrast, MDA concentrations decreased significantly in the INDO + FAM group, while GSH levels increased, although not significantly. This finding is consistent with the study by Soydan et al. using a similar experimental model (Soydan et al., 2025). The administration of Longjing tea reduced MDA levels and increased GSH levels in response to indomethacin-induced damage. These findings suggest that Longjing tea may play an important role against gastric ulcer development by protecting the mucosal structure. This finding is in agreement with previous studies by Maity et al. and Adhikary B. et al. which reported protective effects of tea and tea-derived compounds against gastric ulcer formation (Maity et al., 1995; Adhikary et al., 2011).

Another parameter examined in this study was VEGF-A. VEGF is one of the most important regulators of angiogenesis. It is one of the principal elements that initiate the repair of damage in vital organs by stimulating angiogenesis (Tarnawski et al., 2014). The current literature reports that NSAIDs contribute to the development of ulcers in the mucosa by inhibiting VEGF and angiogenesis, and also to the development of injury by prolonging the healing period (Tarnawski, 2005). Abu-Baih et al. reported that increased oxidative stress following indomethacin administration reduced VEGF levels (Abu-Baih et al., 2024). One of the noteworthy observations in the present study was that indomethacin visibly reduced VEGF-A levels together with rising oxidative stress. This was consistent with previous research (Dudar et al., 2008; Abu-Baih et al., 2024). In addition, VEGF-A levels increased and the healing time was shorter in the group receiving prophylaxis with famotidine, and this was consistent with Abu-Baih et al. (2024). In contrast, Longjing tea administration was associated with reduced VEGF-A levels. This suggested that damage induced with indomethacin may be restored to normal levels with regeneration. Overall, the findings of the present study are consistent with Shaik and Eid (2022). However, Dudar GK. et al. reported that exogenous VEGF may contribute to ulcer healing period in induced gastric ulcers, but not by impacting angiogenesis. Those authors also reported that pathways other than VEGF may independently regulate damage in ulcer pathogenesis (Dudar et al., 2008).

Previous studies have reported an association between VEGF and prostaglandins synthesized via COX enzymes (Antonisamy et al., 2014). The fact that it contributes to the tissue repair by triggering angiogenesis through different signaling pathways, particularly COX, was previously shown by Antonisamy et al. (2016). The COX enzyme has two main forms. COX-1 protects gastric tissue against potential injury caused by gastric acid, in particular, while COX-2 is linked to oxidative stress and trauma arising as a result of cellular trauma (Abdel Fattah and Nasr El-Din, 2025). Indomethacin leads to mucosal and gastric damage by eliminating that protective effect, principally through COX-1 inhibition. As a result, oxidative stress and inflammation increase, leading to mucosal lesions (Rofaeil and Gaber, 2019). Previous studies have also shown that NSAIDs suppress prostaglandin production by inhibiting both COX-1 and COX-2 (Aydin et al., 2025; Ho S, 2025). Consistent with the literature, COX-1 and COX-2 levels in gastric tissue showed a marked but statistically insignificant decrease under in response to indomethacin. A protective effect on COX enzyme levels was observed in the group administered famotidine, suggesting that famotidine may partially counteract the effects of indomethacin. However, the significant increase in COX-1 levels in the group administered Longjing tea constituted evidence that the tea protects the mucosal structure through COX enzymes. Similarly, Adhikary B. et al. previously demonstrated the effects of black tea on indomethacin-induced gastric ulcers and showed that theaflavins exhibited regulatory effects on COX enzymes (Chattopadhyay et al., 2011). In another study, Adhikary B. et al. showed that EGCG, an important component of the *Camellia sinensis* plant and abundantly present in green tea, reverses indomethacin-induced damage to the gastric mucosa (Adhikary et al., 2011). Our finding was again in agreement with the previous literature, with EGCG being identified, as one of the most abundant catechins in the Longjing tea content, responsible for the positive effect that developed.

Reactive oxygen species activity and inflammation triggered in indomethacin-induced gastric injury together lead to the activation of apoptotic mechanisms (Ansari et al., 2025). As deoxyribonucleic acid (DNA) damage is exacerbated by ROS and other factors, PARP-1, a form of the poly ADP-ribose polymerase (PARP) enzyme family, endeavors to prevent damage by protecting the DNA integrity. However, when the damage becomes irreversible, PARP-1 promotes cell death through apoptosis or necrosis (El Latif et al., 2023). He H. et al. previously reported a significant increase in cleaved PARP-1 levels in a toxicity group in their experimental model of ethanol- and aspirin-induced gastric ulcer. However, they observed no significant intergroup difference in total PARP-1 expression levels (He et al., 2019). Similarly, the findings of the present study showed that indomethacin increased cleaved PARP-1 activation, while treatment famotidine and Longjing tea resulted in a reduction of this activation, suggesting a protective effect. Although famotidine exhibited a more powerful protective effect, Longjing tea also demonstrated a considerable antiapoptotic effect in gastric tissue.

Gastric tissues were also subjected to histopathological examination in this study. This revealed a large area of gastric ulcer caused by indomethacin, along with impaired mucosal integrity, increased inflammatory cell infiltration, pronounced hemorrhage in the mucosal area, and submucosal edema. These results were consistent with previous experimental studies (Adhikary et al., 2011; Aly et al., 2024). Similarly to famotidine, the administration of Longjing tea exhibited a protective effect on the mucosa by reducing the ulcer area. This regulatory effect of Longjing tea suggests that it may be caused by its high EGCG content. In line with the experimental ulcer study by Adhikary B. et el. which investigated the effects of EGCG, this compound may contribute to the observed outcomes in the present study (Adhikary et al., 2011).

Another parameter examined in this study was NF-κB/p65. This transcription factor that regulates inflammation and apoptosis and controls multiple signaling pathways (Aboelmagd et al., 2025). Previous experimental studies on indomethacin-induced gastric ulcers have shown a marked increase in NF-κB/p65 levels associated with inflammation and apoptosis, thereby contributing to tissue damage (Aboelmagd et al., 2025; Aydin et al., 2025). Similarly, in the present study, an increase in NF-κB/p65 expression levels was observed, consistent with previous studies. Aydin IC. et al. also reported increased NF-κB/p65 expression, in agreement with our results (Aydin et al., 2025). In contrast, and again consistent with the literature, NF-κB/p65 expression levels decreased in the group administered Longjing tea (Aly et al., 2024; Aboelmagd et al., 2025). These results show that Longjing tea may exhibit its protective effects against indomethacin-induced gastric ulcers at least in part, through modulation of the NF-κB pathway, and may represent a promising candidate for further investigation.

Activation of the PI3K/Akt signaling pathway can regulate the inflammatory response by modulating NF-κB/p65 levels. In addition, Akt is known to play a central role in multiple cellular processes, particularly apoptosis and cell survival downstream of PI3K (Shi et al., 2025). Arab et al. demonstrated an interaction between NF-κB and the PI3K/Akt signaling pathway in an experimental model of ethanol-induced gastric ulcer (Arab et al., 2019). Consistent with previous research, pan-Akt and p-Akt levels in the present study exhibited a partial increase together with NF-κB/p65 in the INDO group. One of the noteworthy findings of this study is that Longjing tea resulted in a moderate, albeit statistically insignificant, decrease in pan-Akt and p-Akt expression. These findings are in line with the study by Xuan F. et al., which demonstrated that EGCG may exert antiapoptotic effects through regulation of the PI3K/Akt signaling pathway in ischemic injury (Xuan and Jian, 2016). However, Zhang X. et al. reported that EGCG can also reduce inflammation by increasing PI3K/Akt signaling (Zhang et al., 2015).

However, the fact that oxidative stress, inflammation, apoptosis, and related signaling pathways have not yet been fully elucidated, and that the partially identified protective effect has not been investigated in a dose-dependent manner, shows that the scope of the current research is limited. It should also be noted that this study represents a preliminary experimental investigation of the effects of Longjing tea.

## 5 Conclusion

This study contributes to the literature, by demonstrating, for the first time, the additive effect of Longjing tea on gastric tissue following indomethacin-induced ulcer formation. These effects were partially mediated by pathways involving COX-1/2, cleaved PARP-1, and PI3K/Akt, important components of NF-κB/p65 signaling. The findings further indicate that Longjing tea exerts partial gastroprotective effects. These effects may be related to the modulation of pathways including COX, NF-κB/p65, and cleaved PARP-1. The results now need to be supported by more extensive studies employing advanced molecular approaches.

## Data Availability

The original contributions presented in the study are included in the article/Supplementary Material, further inquiries can be directed to the corresponding author.
