# Supplementary material for: The synergistic protective effects of bioactive catechins in longjing tea: alleviate indomethacin-induced gastric toxicity through modulation of inflammatory and apoptotic pathways
Source: Front Pharmacol. 2026 May 26;17:1828212. doi: 10.3389/fphar.2026.1828212 (PMC13246347; doi:10.3389/fphar.2026.1828212)
Supplement: Supplementary file 1 [file Supplementaryfile1.pdf]

Supplementary material:

CHROMATOGRAM

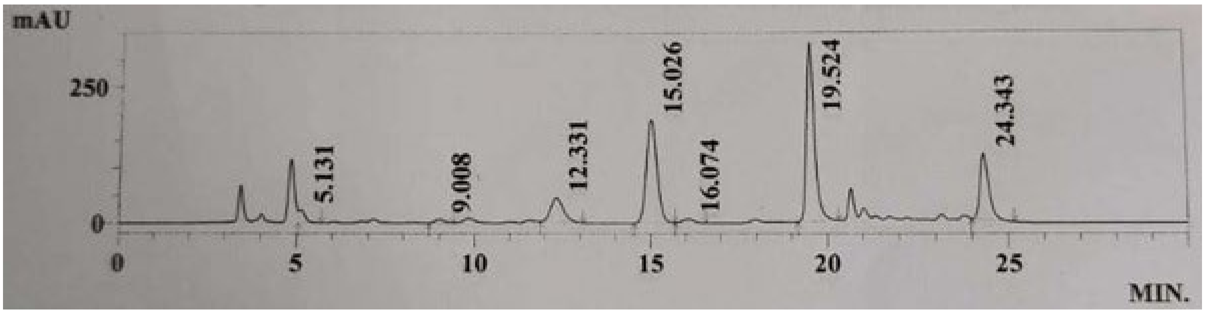

PDA Multi 1/ 278nm 4 nm

QUANTİTATİVE RESULTS

PDA

ID#	NAME	RET.TIME	AREA	CONC.	UNITS
1	**Gallic acid**	**5.131**	**285415**	**8.237**	**ppm**
2	**Epigallocatechin (EGC)**	**9.008**	**102923**	**37.156**	**ppm**
3	**Catechin (C)**	**12.331**	**1087192**	**564.740**	**ppm**
4	**Caffeine**	**15.026**	**3922154**	**118.957**	**ppm**
5	**Epicatechin (EC)**	**16.074**	**140106**	**15.834**	**ppm**
6	**Epigallocatechin gallate (ECGG)**	**19.524**	**51008114**	**344.298**	**ppm**
7	**Epicatechin gallat**	**24.343**	**2009933**	**91.296**	**ppm**

TOTAL POLYPHENOL

**The dry matter**	**93.28**
**Total catechin content**	**32.18%**
**Average**	**32.10±6.03%**
**Total polyphenols**	**22.30±2.01%**
